# Cardiac-specific knockdown of Bhlhe40 attenuates angiotensin II (Ang II)-Induced atrial fibrillation in mice

**DOI:** 10.3389/fcvm.2022.957903

**Published:** 2022-10-11

**Authors:** Kai-Wen Ren, Xiao-Hong Yu, Yu-Hui Gu, Xin Xie, Yu Wang, Shi-hao Wang, Hui-Hua Li, Hai-Lian Bi

**Affiliations:** ^1^Institute of Cardiovascular Diseases, First Affiliated Hospital of Dalian Medical University, Dalian, China; ^2^Department of Cardiology, First Affiliated Hospital of Dalian Medical University, Dalian, China; ^3^Department of Emergency Medicine, Beijing Key Laboratory of Cardiopulmonary Cerebral Resuscitation, Beijing Chaoyang Hospital, Capital Medical University, Beijing, China

**Keywords:** atrial fibrillation, atrial inflammation, atrial fibrosis, Bhlhe40, NLRP3

## Abstract

Atrial fibrosis and atrial inflammation are associated with the pathogenesis of atrial fibrillation (AF). Basic helix–loop–helix family member E40 (Bhlhe40) is an important transcription factor, which is involved in tumors, inflammation, apoptosis, viral infection, and hypoxia. However, its role and molecular mechanism in AF remain unclear. In this study, a mouse model of AF was induced by Ang II infusion. The atrial diameter was evaluated using echocardiography. Induction and duration of AF were measured by programmed electrical stimulation. Atrial structural remodeling was detected using routine histologic examinations. Our results showed that Bhlhe40 was significantly upregulated in angiotensin II (Ang II)-stimulated atrial cardiomyocytes and atrial tissues and in tissues from patients with AF. Cardiac-specific knockdown of Bhlhe40 in mice by a type 9 recombinant adeno-associated virus (rAAV9)-shBhlhe40 significantly ameliorated Ang II-induced atrial dilatation, atrial fibrosis, and atrial inflammation, as well as the inducibility and duration of AF. Mechanistically, cardiac-specific knockdown of Bhlhe40 attenuated Ang II-induced activation of NF-κB/NLRP3, TGF-1β/Smad2 signals, the increased expression of CX43, and the decreased expression of Kv4.3 in the atria. This is the first study to suggest that Bhlhe40 is a novel regulator of AF progression, and identifying Bhlhe40 may be a new therapeutic target for hypertrophic remodeling and heart failure.

## Introduction

Atrial fibrillation (AF) is the most common sustained cardiac arrhythmia, with considerable morbidity and mortality, and it increased the risk of embolic stroke and heart failure. Current pharmacological strategies for AF have limited effectiveness and significant adverse-effect potential, and also have high recurrences and potential complications for AF ablation (1, 2). Thus, a better understanding of the mechanisms in the initiation and progression of AF and identifying new targets are needed for creating and exploiting novel therapeutic avenues (2).

Atrial tissue fibrosis is a main factor of the persistence and progression of AF and a central pathophysiological feature of AF (3). The renin–angiotensin system (RAS) hormone angiotensin II (Ang II) plays an important role in the initiation and development of AF (4). Increasing evidence has demonstrated that the Ang II cell membrane receptor, mainly angiotensin II type 1 receptor (AT1R), which *via* multiple signaling pathways, such as transforming growth factor-β (TGF-β)/mothers against decapentaplegic 2/3 (Smad2/3) and NACHT, LRR, and PYD domains-containing protein-3 (NLRP3)/nuclear factor kappa-B (NF-κB), regulate fibrosis formation and inflammation, and play a prominent role in AF (5). Recently, studies demonstrated that cardiomyocyte (CM) NLRP3 inflammasome activation and NF-κB activation are key proarrhythmic mediators of multiple pathophysiological signals in AF and have direct effects on atrial fibrosis, ion channel, and connexin dysfunction in mouse and rabbit atria (4, 6, 7). Thus, blocking the NLRP3 inflammasome activation and/or NF-κB activation helps prevent AF progression.

Bhlhe40 (also called DEC1, stra13, and sharp2) is one of the basic helix–loop–helix (bHLH) transcriptional factors containing three domains, including, a basic DNA-binding domain (DBD), an HLH domain mediating dimerization, and orange domain, which is a domain-mediated protein–protein interaction. It can directly bind to DNA at E-box DNA response elements and functions primarily as a transcriptional repressor (8, 9). Previous studies indicate that Bhlhe40 plays a critical role in several pathological conditions, including tumors, inflammation, apoptosis, and hypoxia. It has been reported that it controls cytokine production, including IL-10, GM-CSF, and other cytokines, in T cells during infection and autoimmunity (10–12). Recently, a study using single-cell RNA sequencing has reported that the expression of Bhlhe40 is increased in atrial cardiac fibroblasts (ACFs) derived from patients with AF (13). However, the function of Bhlhe40 in cardiac arrhythmia, particularly in Ang II-induced AF, has not been investigated.

In this study, we explored the expression and effects of Bhlhe40 on the maintenance and progression of AF in an Ang II-induced mice model. The expression of Bhlhe40 was significantly increased both in Ang II-induced atrial tissues in mice and in the atrial tissues from patients with AF. Meanwhile, cardiac-specific Bhlhe40-knockdown mice showed significantly attenuated susceptibility to AF, and repressed atrial structural and electrical remodeling in Ang II-infused mice. Furthermore, the mechanistic study showed that the protective effect of knockdown of Bhlhe40 is associated with the inhibition of multiple signaling pathways (TGF-β1/Smad2, NF-κB/NLRP3, and the expression of Kv4.3 and CX43). Collectively, our results suggest that Bhlhe40 contributes to atrial remodeling and may be a novel target for the treatment of hypertensive AF.

## Materials and methods

### Study subjects

A total of 49 patients with proven AF (paroxysmal or persistent AF) were recruited, who underwent catheter ablation for the first time from March 2021 to March 2022. In this study, the control subjects (*n* = 35) with normal sinus rhythm, without any family history of AF, and no obvious abnormalities in physical examination (routine examination, clinical examination, laboratory reports, and B-ultrasonography reports) were also recruited. Exclusion criteria are as follows: patients with ventricular structural remodeling (EF < 50% or BNP > 500 ng/ml), myocardial structural lesions, diabetes, stroke, thyroid disease, hematologic system diseases, surgery, or trauma within 2 years, acute or chronic infectious disease, cancer, and significant renal dysfunction (estimated glomerular filtration rate < 30 ml/min per 1.73 m^2^). Before catheter ablation, a series of blood samples were collected from SR controls or patients with AF for ELISA. Moreover, among the SR controls and patients with AF, we only obtained atrial tissues from seven SR controls and patients with AF, respectively, which are prepared for histologic examinations. Patients who participated in this study gave their informed consent, and the study was approved by the Ethics Committee of the First Affiliated Hospital of Dalian Medical University.

### Animal experiments

For this experiment, 2-month-old male C57BL/6 mice purchased from the Jackson Laboratory (Bar Harbor, ME, USA) were anesthetized with an intraperitoneal injection of 2.5% tribromoethanol (0.02 ml/g; Sigma-Aldrich, St. Louis, MO, USA) and infused with Ang II (2,000 ng/kg/min, A107852; Aladdin, Shanghai, CHN) or saline using osmotic mini-pumps (Alzet model 1004; Durect, Cupertino, CA, USA) for 3 weeks, as previously described (14). This entire study was approved by the Institutional Ethics Committee of the First Affiliated Hospital of Dalian Medical University (No. LCKY2016-31) and conformed to the Guide for the Care and Use of Laboratory Animals published by the U.S. National Institutes of Health (NIH Publication No. 85-23, revised 1996).

### Isolation and culture of neonatal rat atrial cardiomyocytes

The atria were isolated from the hearts of the 1- to 3-day-old Sprague–Dawley rats and digested in trypsin (25200056, Thermo Fisher, Waltham, MA, USA) at 37°C for 6–8 cycles. The cells were collected and incubated for 90 min at 37°C. Then, cell suspension was collected and centrifuged at 1,000 rpm for 10 min, and then cultured in DMEM/F12 (SH30023.01B, Hyclone, South Logan, UT, USA) with 10% FBS (16140071, Gibco, Grand Island, NY, USA), 1% penicillin/streptomycin, and 100 mM BrdU. After 36-h incubation, the cells were serum-starved for 12 h and then treated with Ang II (100 nM) or saline for 24 h.

### Injection of recombinant adeno-associated virus

Recombinant adeno-associated virus serotype 9 (rAAV9) vectors containing short hairpin RNAs (shRNAs) targeted at Bhlhe40 (rAAV9-shBhlhe40) or a control scramble sequence (rAAV9-shCON) were used, which were purchased from Vigene Biosciences. The sequences were used as follows: siRNA1, GGAGAACGTGTCAGCACAAT; siRNA2, GCCTTCCCTTCTATCTCAT; siRNA3, GGATCTCCTACCCGAACATCT; and siRNA4, GCGGTTTACAAGCTGGTGAT. The shRNA expression 4 in 1 was driven by a mouse U6 promoter (pol III), followed by the reporter sequence (GFP). The final content of the virus (2 × 10^11^ vg) was diluted in normal saline (saline).

### Blood pressure measurement

Measurements of systolic blood pressure (SBP) were taken from the beginning of Ang II or saline infusion and every 3 days after infusion using a tail-cuff system (BP-2010, Softron, Tokyo, JPN), as reported previously (15).

### Echocardiographic measurement

The mice were anesthetized with 1.5% isoflurane (Sigma-Aldrich). An echocardiographic measurement of the left atrial diameter of the mice was performed using a Vevo 2100 High-Resolution Imaging System (Visual Sonics, Inc, Toronto, Ontario, Canada), as reported previously (15).

### Induction and duration of atrial fibrillation

The mice were anesthetized with an intraperitoneal injection of 2.5% tribromoethanol at a dose of 0.02 ml/g (Sigma-Aldrich, St. Louis, MO, USA). An electric blanket waterproof heating pad for mice (JRD-7w; Nomoy Pet, Jiaxing, China) was used to maintain the body temperature of the mice at about 37°C. A recording/stimulation electrophysiology catheter, a Millar 1.9F octapolar electrophysiology catheter (Scisense, London, Ontario, Canada), was inserted into the right jugular vein and then into the right atrium to achieve the intracardiac pacing. Burst pacing containing 33 impulses for each individual mouse, at different voltage magnitudes and different frequencies, was used to induce AF. After burst pacing with different impulses, the duration of the AF was recorded, as reported previously (16). AF was defined as rapid (atrial rates > 1,500 beat/min) and irregular atrial episodes lasting ≥ 0.5 s.

### Histological analysis

Atrial samples from the patients with AF or mice were removed and fixed in 4% paraformaldehyde for more than 2 days and then embedded in paraffin. Serial sections of 4 μm thickness were cut and stained with an H&E staining kit (Nanjing Jiangcheng Bio Inc., Nanjing, China) and a Masson staining kit (Nanjing Jiangcheng Bio Inc., Nanjing, China). The quantification of the areas of fibrosis was measured by ImageJ software. Immunohistochemistry (IHC) was incubated with indicated first antibodies: anti-Bhlhe40 (1:100; 17895-1-AP), anti-α-SMA (1:200; 67735-1-Ig), anti-NLRP3 (1:200; 19771-1-ap) (Proteintech, Wuhan, HB, China), or anti-F4/80 (1:100; ab6640, Abcam, Cambridge, MA, USA) overnight at 4°C and then incubated with appropriate secondary antibodies for 30 mins at 37°C and detected using a DAB Kit (E-IR-R217, Elabscience Biotechnology Co., Ltd, Wuhan, China). Quantitative analysis of histologic staining was performed by ImageJ, as previously described (17).

### Immunofluorescence

The sections were blocked in blocking buffer (Triton X-100, 0.3M glycine, and 1% BSA) for 30 min at room temperature, and then the sections were incubated with anti-CX43 (1:100, 26980-1-AP, Proteintech) or anti-F4/80 (1:100; ab6640, Abcam, Cambridge, MA, USA) overnight at 4°C in PBS and washed with PBS. The sections were incubated with the secondary antibodies (A0453, Beyotime) for 30 mins at 37°C. Nuclear DNA was labeled with DAPI. The image was taken with a 63x objective on a Leica STELLARIS 5 confocal microscope. The quantification of the intensity of CX43-positive cells was measured by ImageJ software.

### Quantitative real-time PCR analysis

Total RNA from each atrial tissue was extracted using TRIzol (Invitrogen/Thermo Fisher Scientific, Carlsbad, CA, USA) and then was used to synthesize the first-strand cDNA using Random Primers/Oligo (dT)-primer mix RT kits (11141ES60; Yeasen, Shanghai, China) according to the manufacturer’s protocol. The mRNA levels of Bhlhe40, the fibrotic marker collagen I (Col I) and collagen III (Col III), and the inflammatory markers IL-1β and IL-6 were determined by real-time PCR (qPCR) analysis using SYBR Green (RR820A, Takara Bio Inc. Shiga, Japan) on a 7500 Real-Time PCR System (Applied Biosystems, USA). The relative expression of each gene was normalized to glyceraldehyde-3-phosphate dehydrogenase (GAPDH). Primers were purchased from Sangon Biotech (Shanghai, China). Primer sequences are provided in Table 1.

**TABLE 1:** Primers used in qPCR.

Gene	Forward primer	Reverse primer
Bhlhe40	TGGTGATTTGTCGGGAAGAAA	ACGGGCACAAGTCTGGAAAC
collagen I	AGTCGATGGCTGCTCCAAAA	AGCACCACCAATGTCCAGAG
collagen III	TCCTGGTGGTCCTGGTACTG	AGGAGAACCACTGTTGCCTG
IL-1β	TGAAAACACAGAAGTAACGTCCG	CCCAGGAGGAAATTGTAATGGGA
IL-6	TTCCATCCAGTTGCCTTCTTG	TTGGGAGTGGTATCCTCTGTGA

### Immunoblotting analysis

Protein lysates, extracted from atrial tissues, were lysed with radioimmunoprecipitation assay (RIPA) buffer (Solarbio Science Technology Co., Beijing, China) and then concentrated using a BCA protein assay. The same amount of protein (40–60 μg) was electrophoresed on 8–12% polyacrylamide gels and transferred to polyvinylidene difluoride membranes (Bio-Rad), which was incubated with primary antibodies. The antibodies against Bhlhe40 (1:100; 17895-1-AP), TGF-β1 (1:1,000; 21898-1-AP), CX43 (1:1,000; 26980-1-AP), NLRP3 (1:1,000; 19771-1-ap), and tubulin (1:3,000, 66031-1-Ig) were purchased from Proteintech (Wuhan, HB, China). Smad2 (1:1,000; 5339S) and p65 (1:1,000; 4764S) were bought from Cell Signaling Technologies (Boston, MA, USA). The antibodies phosphorylated (p)-Smad2 (1:1,000; ab280888) and Kv4.3 (1:1,000; ab65794) were obtained from Abcam. p-p65 (1:500; 310013) antibody was purchased from Zen Bioscience (Chengdu, Sichuan, China). HRP-labeled secondary antibodies were bought from Beyotime (A0208, A0216). All blots were detected, analyzed, and normalized to tubulin.

### Enzyme-linked immunosorbent assay

In brief, the samples consisted of 49 serum samples obtained from individuals with AF and 35 serum samples from individuals with SR. Then, serum levels of total Bhlhe40 were determined using human sharp2/Bhlhe40 ELISA kits (FS204153; Westang Bio Inc., Shanghai, China) according to the manufacturer’s instructions.

### Statistical analysis

Statistical calculations were analyzed using GraphPad Prism 8.0. A normality test was conducted first. If all the groups satisfied the normality criteria and variances between the groups were equal, we applied Student’s *t*-test or one-way ANOVA (with the Bonferroni *post hoc* test), where appropriate; if the aforementioned conditions were not met, we used the nonparametric Mann–Whitney U test. Statistical differences were determined by *p < 0.05*. Results are presented as mean ± SD.

Univariable logistic regression and multivariable logistic regression were used to investigate possible factors associated with AF. A total of three models were analyzed: model 1, the crude model without covariate adjustment; model 2, the multivariable model that adjusted for sex and age, and model 3, the full risk adjustment model that adjusted potential confounders that were significant on univariate analysis and those known to be associated with AF, including sex, age, LA diameter, total cholesterol (TC), and HDL cholesterol.

## Results

### Expression of Bhlhe40 is upregulated in patients with atrial fibrillation

**FIGURE 1:**
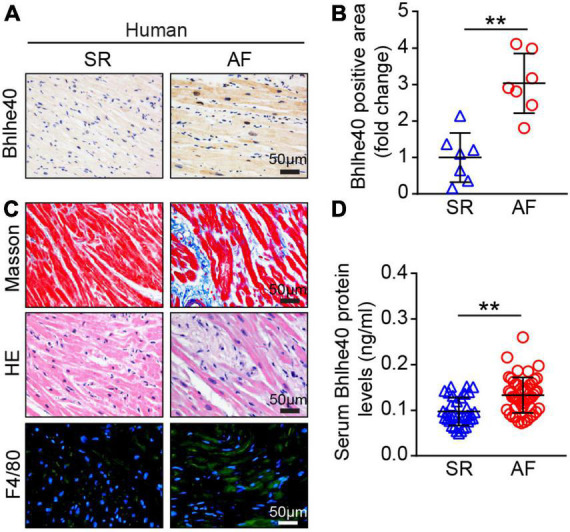
Expression of Bhlhe40 was increased in patients with AF. **(A,B)** Immunohistochemical (IHC) staining of Bhlhe40 expression in atria from sinus rhythm (SR) controls and patients with AF (left), and quantification of the Bhlhe40-positive area (brown color; *n* = 7, left). Scale bar = 50 μm. **(C)** Masson staining (upper) of atrial sections for examination of fibrosis (blue); H&E staining (middle) of atrial sections for detection of inflammatory cell infiltration (blue), and immunofluorescent staining (lower) of atrial sections for examination of F4/80-positive macrophages (green) and nuclei (DAPI, blue) (*n* = 7). Scale bar = 50 μm. **(D)** ELISA of the serum Bhlhe40 level from SR controls (*n* = 35) and patients with AF (*n* = 49). **P < 0.01 vs normal SR controls.

To identify the changes of Bhlhe40 in the progression of AF, we detected the expression of Bhlhe40 both in patients with AF and sinus rhythm (SR). Immunohistochemical staining indicated that the Bhlhe40-positive area was significantly increased in atrial tissues from patients with AF compared with sinus rhythm (SR) controls (Figures 1A,B). Meanwhile, Masson, H&E, and immunofluorescent staining revealed that the degree of atrial fibrosis and infiltration of inflammatory cells, including F4/80-positive macrophages, were obviously higher in atrial tissues from patients with AF than in SR controls (Figure 1C). To test whether Bhlhe40 has a critical role in human AF, we further examined the serum Bhlhe40 level in patients with AF. ELISA showed that the mean serum Bhlhe40 level was significantly higher in patients with AF than in the SR control (*P*<0.001) (Figure 1D). We also found that the patients with AF had a higher LA diameter and total cholesterol and lower LDL cholesterol than the sinus rhythm (SR) control (*P*<0.05) (Table 2). We then evaluated the association of AF and blood (serum or plasma) Bhlhe40 with both univariable and multivariable logistic regression models (Table 3). After being adjusted for the aforementioned confounding factors in model 3, we found that Bhlhe40 is causally associated with AF. Specifically, the odds ratio (OR) of AF per 1 standard deviation (SD) increase in Bhlhe40 is estimated to be 2.763 [95% CI, 1.238, 6.169; *P = 0.013*], suggesting that the upregulation of Bhlhe40 may play a role in the development of AF.

**TABLE 2:** Clinical characteristics.

Parameters	SR (*n* = 35)	AF (*n* = 49)	*P*-value
**Demographics**			
Male, *n* (%)	62.9	61.2	
Age, y	61.1 ± 8.3	64.4 ± 11.4	0.151
**Hemodynamic variables**			
Resting heart rate, beats/min	76.5 ± 11.0	79.3 ± 17.8	0.412
SBP, mmHg	136.7 ± 13.2	135.1 ± 16.0	0.624
DBP, mmHg	90.2 ± 9.2	89.5 ± 10.7	0.728
BMI, kg/m^2^	24.1 ± 3.2	25.4 ± 4.3	0.140
**Echocardiography**			
LVEF, %	56.2 ± 1.7	57.3 ± 3.2	0.054
LA diameter, mm	35.9 ± 2.6	39.4 ± 3.7	<0.001**
**Blood-based biomarkers**			
Bhlhe40, ng/ml	0.097 ± 0.031	0.133 ± 0.040	<0.001**
FBG, mmol/L	5.4 ± 1.1	5.2 ± 0.9	0.302
AST, umol/L	21.1 ± 7.0	48.2 ± 194.0	0.334
SCr, umol/L	72.3 ± 13.6	76.5 ± 27.9	0.358
TC, mmol/L	5.1 ± 1.1	4.3 ± 1.3	0.002**
LDL, mmol/L	2.8 ± 0.8	2.3 ± 1.0	0.010*
HDL, mmol/L	1.16 ± 0.3	1.13 ± 0.4	0.636
BNP, pg/ml	NA	260.24 ± 308.21	

*P < 0.05, **P < 0.01 vs SR control.

SR, sinus rhythm; AF, atrial fibrillation; SBP, systolic blood pressure; DBP, diastolic blood pressure; LA, left atrial; LVEF, left ventricular ejection fraction; FBG, fasting blood glucose; AST, aspartate transaminase; SCr, serum creatinine; TC, total cholesterol; and LDL, low-density lipoprotein; HDL, high-density lipoprotein; NA, not applicable; and. BNP indicates B-type natriuretic peptide. The parameters are mean (SD) or *n* (%).

**TABLE 3:** Multiple logistic regression analysis of Bhlhe40 associated with AF.

Bhlhe40, per SD increment	OR (95%CI)	*P*-value
Model 1	3.381 (1.799, 6.353)	<0.001
Model 2	3.292 (1.745, 6.208)	<0.001
Model 3	2.763 (1.238, 6.169)	0.013

Model 1 was unadjusted, model 2 was adjusted for sex and age, and model 3 was adjusted for all potential confounders, including sex, age, LA diameter, total cholesterol (TC), and LDL cholesterol.

### Enhanced expression of Bhlhe40 is found in Ang II-induced atrial tissue

**FIGURE 2:**
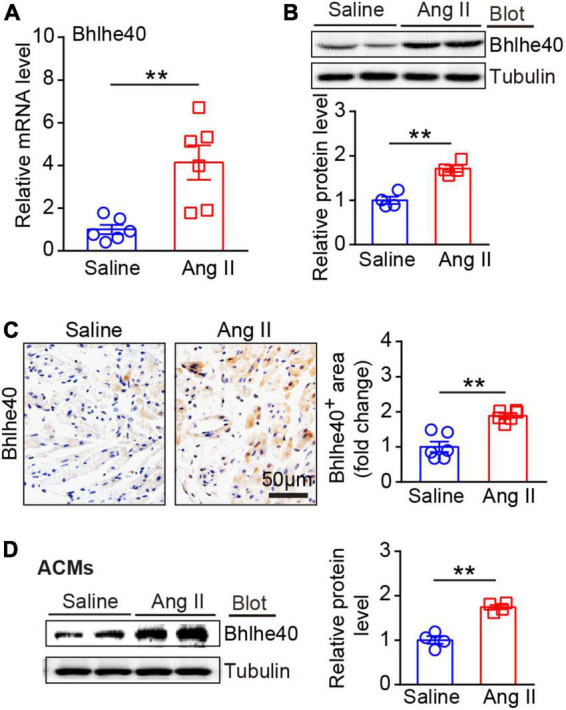
Expression of Bhlhe40 was significantly upregulated in Ang II-induced atrial tissues and atrial myocytes. **(A)** qPCR analysis of the mRNA level of Bhlhe40 in Ang II-infused or saline-infused atrial tissues (*n* = 6). **(B)** Immunoblotting analysis of Bhlhe40 expression in atrial tissues from each group (upper), and quantification of the Bhlhe40 level (lower, *n* = 4). **(C)** IHC staining of Bhlhe40 (left) and the quantification of the Bhlhe40-positive area (right) in atrial tissues from Ang II-infused or saline-infused mice (*n* = 6). Scale bar = 50 μm. **(D)** primary atrial cardiomyocytes (ACMs) were treated with Ang II (100 nM) or Saline for 24 h. Bhlhe40 protein levels in ACMs were detected by immunoblotting analysis (left) and quantified (right) (*n* = 4). **P < 0.01 vs saline-treated WT mice.

Next, we detected the changes of Bhlhe40 expression in Ang II-infused WT atrial tissues. After 21 days of Ang II or saline infusion, qPCR and immunoblotting analysis showed that Bhlhe40 expression at both mRNA and protein levels was significantly elevated in Ang II-infused atrial tissues compared with saline-treated controls (Figures 2A,B). Furthermore, immunohistochemical staining further demonstrated the increased expression of Bhlhe40 in Ang II-infused atrial tissues (Figure 2C). Similarly, a significant increase in the protein level of Bhlhe40 was also observed in cultured atrial cardiomyocytes (ACMs) treated with Ang II (Figure 2D). Thus, the increased expression of Bhlhe40 indicated that Bhlhe40 in ACMs may participate in the development of AF.

### Cardiac-specific knockdown of Bhlhe40 attenuates Ang II-induced atrial remodeling and atrial fibrillation inducibility in mice

**FIGURE 3:**
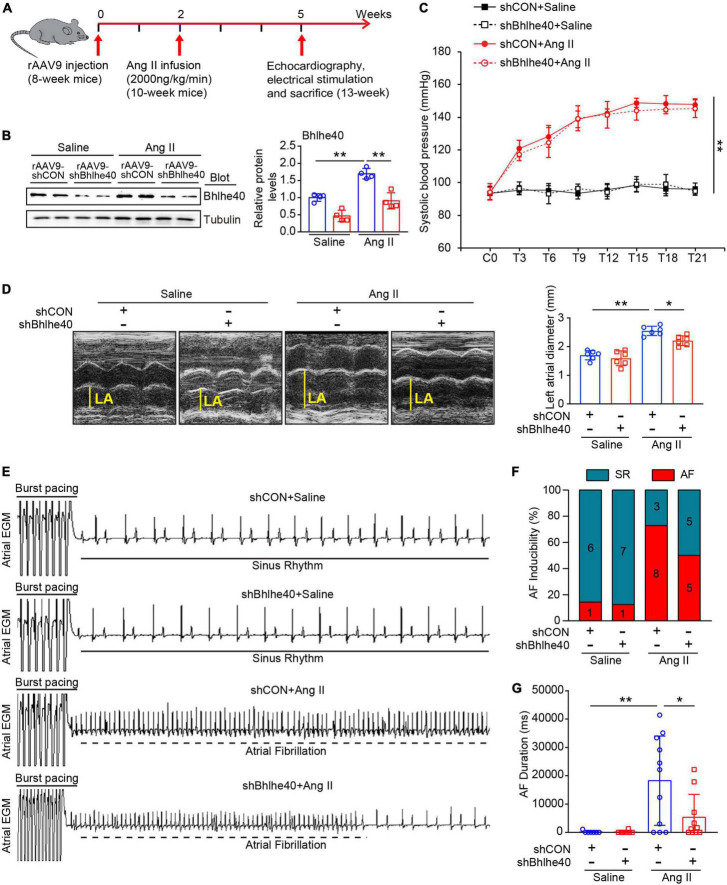
Knockdown of Bhlhe40 by rAAV9-shBhlhe40 injection attenuates Ang II-induced atrial remodeling and AF in mice. **(A)** Protocol for injection of rAAV9 in mice of cardiac remodeling. Mice were injected with rAAV9-shBhlhe40 or rAAV9-shCON for 2 weeks and then treated with Ang II (2,000 ng/kg/min) infusion for additional 3 weeks. **(B)** Immunoblotting analysis of Bhlhe40 expression in mice injected with rAAV9s and infused with Ang II or saline (left). The quantification of the Bhlhe40 level (right, *n* = 4). **(C)** Systolic blood pressure (SBP) was measured by the tail-cuff method every 3 days after Ang II administration (*n* = 6). **(D)** Echocardiographic measurement LA dilation (left) and the quantification (right) in Ang II or saline-infused mouse (*n* = 6). **(E)** Representative atrial electrogram recordings for AF or SR control. The solid lines indicate burst pacing, and the dashed lines indicate AF. **(F)** Percentage of mice with AF in each group (*n* = 7–11). **(G)** Total AF duration for each rAAV9-shCON-injected and rAAV9-shBhlhe40-injected mouse treated with Ang II or saline infusion. *P < 0.05, **P < 0.01.

To determine the role of Bhlhe40 in AF development, we generated recombination adenoassociated virus type 9 (rAAV9)-expressing shBhlhe40 to selectively knock down endogenous Bhlhe40 expression in cardiomyocytes (CMs). The wild-type (WT) mice were injected with rAAV9-shBhlhe40 or its control vector (rAAV9-shCON) for 2 weeks and then treated with Ang II (2,000 ng/kg/min) infusion for additional 3 weeks to induce AF (Figure 3A). The efficiency and specificity of rAAV9-shBhlhe40 knockdown in the atria were confirmed by immunoblotting assay and IHC staining (Figure 3B and Supplementary Figure 1A). We found that rAAV9-shBhlhe40 injection significantly reduced the Bhlhe40 protein level by 30% in the atrial tissues, by IHC staining, but not in lungs and skeletal muscles, compared with rAAV9-shCON injection (Supplementary Figures 1A–C). Thus, these data indicate that rAAV9-shBhlhe40 injection selectively knocks down endogenous Bhlhe40 expression in ACMs. Moreover, following Ang II infusion, both rAAV9-shCON (control)- and rAAV9-shBhlhe40-injected mice had elevated systolic blood pressure (SBP) compared with baseline levels, but the cardiac-specific knockdown of Bhlhe40 did not affect SBP elevations caused by Ang II infusion (Figure 3C). Since atrial structural remodeling is a central pathophysiological feature of AF, we aimed to examine the effect of Bhlhe40 on atrial dilation by using echocardiography. Ang II-induced elevation of the dilatation of the left atrium (LA) was significantly reduced in the rAAV9-shBhlhe40 group (Figure 3D).

To further assess the role of Bhlhe40 in the development of AF, we examined the vulnerability to AF by programmed electrical stimulation (Figure 3E), as measured by the inducibility and duration of Ang II-infused AF in rAAV9-shBhlhe40- or rAAV9-shCON-injected mice. The inducibility of AF was obviously increased in Ang II-infused mice both in the rAAV9-shCON group and rAAV9-shBhlhe40 group compared with the saline-infused mice, respectively (Figure 3F, 72.7 versus 14.3%, and 50 versus 12.5%). However, the knockdown of Bhlhe40 reduced Ang II-triggered AF inducibility (50 versus 72.7%). The total duration of AF was also preserved in the Ang II-infused rAAV9-shBhlhe40 group (Figure 3G). In addition, the heart rates of the mice in all groups were not significantly changed between the rAAV9-shBhlhe40 group and rAAV9-shCON group with saline or Ang II infusion. There is no significant difference between the rAAV9-shCON group and rAAV9-shBhlhe40 group (Figures 3C–G).

### Cardiac-specific knockdown of Bhlhe40 attenuates Ang II-induced fibrosis

**FIGURE 4:**
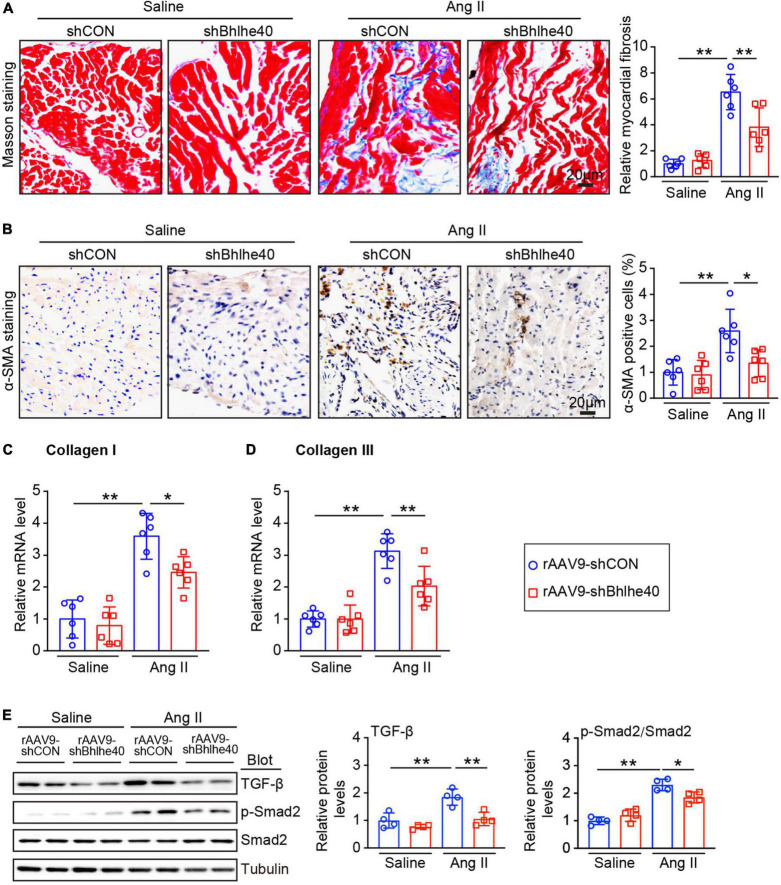
Knockdown of Bhlhe40 by rAAV9-shBhlhe40 injection inhibits Ang II-induced atrial fibrosis. **(A)** Representative Masson staining for fibrosis that stained in blue (left) and quantification of the fibrotic area of the Masson-stained sections from the rAAV9-shCON-treated/rAAV9-shBhlhe40- treated and Ang II-infused/saline-infused hearts (right; *n* = 6 mice per group). **(B)** Representative IHC and quantification of α-SMA-positive cells (right; *n* = 6 mice per group). **(C,D)** qPCR analyses of collagen I and collagen III mRNA levels in Ang II-induced atrial tissues. **(E)** Immunoblotting analyses of TGF-β1, p-Smad2, and Smad2 protein levels in atrial tissues (left), and the quantification of each protein (right, *n* = 4). *n* indicates the number of animals used in each group. *P < 0.05, **P < 0.01.

We then determined the effect of Bhlhe40 on atrial fibrosis, a central pathophysiological feature and a main factor of AF. As shown by Masson staining, the atrial fibrotic area augmented in Ang II-infused mice compared with saline infused mice whereas knockdown of Bhlhe40 significantly attenuated this effect (Figure 4A). Furthermore, immunohistochemical staining revealed the Ang II-induced increase in the number of α-SMA-positive cells was markedly attenuated in the rAAV9-shBhlhe40-injected mice compared with that in the rAAV9-shCON-injected mice (Figure 4B). In addition, the Ang II-induced elevation of the fibrotic markers (collagen I and III) at the transcriptional level was also reduced in the rAAV9-shBhlhe40-injected mice (Figures 4C,D).

As our preceding data showed that knockdown of Bhlhe40 attenuates Ang II-induced AF, we next determined whether the knockdown of Bhlhe40 could reduce the fibrosis-related signaling pathways in Ang II-induced AF. Ang II-induced activation of TGF-1β/Smad2/3 signals in the atria of the rAAV9-shCON-injected mice was markedly reversed in the rAAV9-shBhlhe40-injected mice (Figure 4E). Together, these results suggest that the knockdown of Bhlhe40 inhibits Ang II-induced fibrosis and atrial remodeling.

### Cardiac-specific knockdown of Bhlhe40 attenuates Ang II-induced electrical remodeling

**FIGURE 5:**
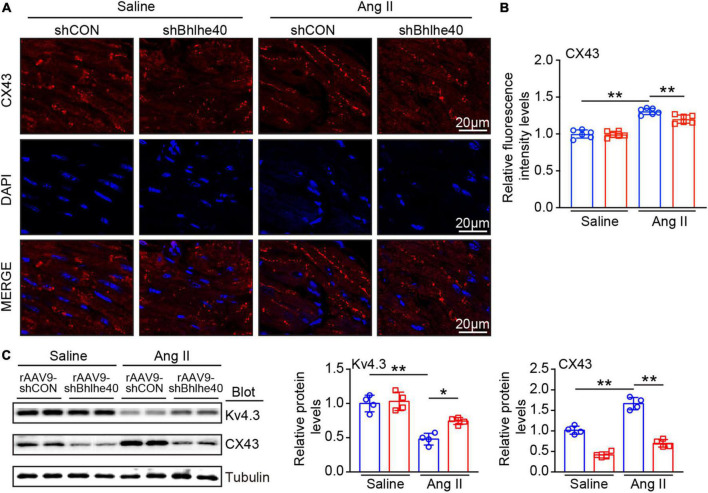
Knockdown of Bhlhe40 by rAAV9-shBhlhe40 injection reduces Ang II-induced atrial electrical remodeling. **(A)** Representative immunofluorescence for Cx43 (red) in atrial tissues from Ang II-infused or saline-infused mice. **(B)** Quantification of the intensity of CX43 expression in atrial tissues. **(C)** Immunoblotting analyses (left) and quantification of ion channel subunits and connexin (Kv4.3and CX43) in atrial tissues (*n* = 4). Tubulin was used as an internal control. *n* represents the number of animals (*n* = 4). *P < 0.05, **P < 0.01.

We further assessed whether the loss of Bhlhe40 reduced vulnerability to AF by regulating the distribution and expression of ion channel and gap junction proteins in atrial tissues (18). Immunofluorescence staining for CX43 (red) was regular and mainly located at the cell poles in the saline-infused group, while the pattern in the Ang II-infused group was irregular, heterogeneous, and located both at the cell poles and at the lateral side of the cells (Figures 5A,B). Moreover, the expression of Kv4.3 (K^+^ channel, potassium voltage-gated channel subfamily D member 3) induced by Ang II infusion was decreased in the atria of the rAAV9-shCON-injected mice but was reversed in the Bhlhe40-knockdown mice (Figure 5C). Meanwhile, the increased protein level of CX43 (connexin 43), induced by Ang II infusion, was markedly reversed in the Bhlhe40-knockdown mice (Figure 5C). There was no significant change in CX43 and Kv4.3 expression between the rAAV9-shCON- and rAAV9-shBhlhe40-injected mice after saline or Ang II infusion (Figures 5A–C).

### Cardiac-specific knockdown of Bhlhe40 attenuates Ang II-induced inflammation

**FIGURE 6:**
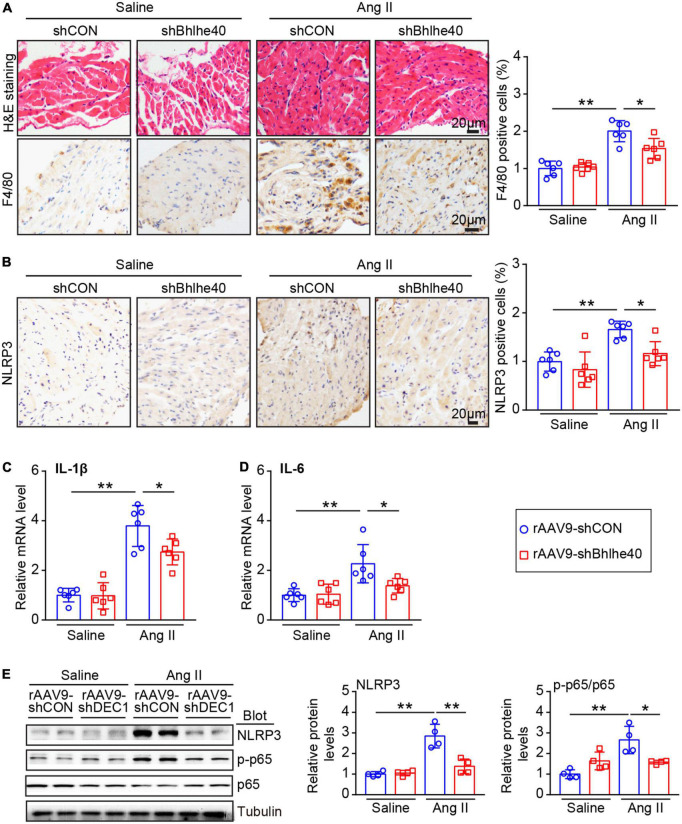
Knockdown of Bhlhe40 by rAAV9-shBhlhe40 injection suppresses Ang II-induced atrial inflammation. **(A)** Representative hematoxylin–eosin (H&E) staining (top in left), IHC staining of F4/80 (bottom in left), and the quantification of F4/80-positive cells (right) from the IHC-stained sections from the rAAV9-shCON/rAAV9-shBhlhe40-treated and Ang II/saline-infused atrial tissues (*n* = 6 mice per group). **(B)** Representative images of NLRP3 immunohistochemistry (left) and the quantification of NLRP3-positive cells (right; *n* = 6 mice per group). **(C,D)** qPCR analyses of pro-inflammatory cytokine levels (IL-1β and IL-6) in atrial tissues (*n* = 6). **(E)** Immunoblotting analyses (Left) and quantification of ion channel subunits and connexin (Kv4.3and CX43) in atrial tissues (*n* = 4). Tubulin served as an internal control. *n* indicates the number of animals. *P < 0.05 and **P < 0.01.

According to reports, the activation of cardiomyocyte NLRP3 inflammasomes is common in AF and is associated with atrial fibrosis and electrical remodeling (6, 7). We further assessed whether cardiac-specific knockdown of Bhlhe40 influences the inflammatory response and the NLRP3 inflammasome pathway in the atria. The rAAV9-shCON-injected mice showed a significant increase in inflammatory cell infiltration after Ang II injection, including F4/80-positive macrophages, but this increase was attenuated in the rAAV9-shBhlhe40-injected mice (Figure 6A). Moreover, the Ang II-induced increase in NLRP3 expression observed in the rAAV9-shCON-injected mice was decreased in the rAAV9-shBhlhe40-injected mice, as measured by immunohistochemistry (Figure 6B). In addition, the mRNA levels of IL-1β and IL-6 (pro-inflammatory cytokines and downstream targets of NLRP3 inflammasome activation) was downregulated in atrial tissue from the rAAV9-shBhlhe40-injected group compared with the rAAV9-shCON-injected group that treated with Ang II infusion (Figures 6C,D).

NLRP3 and/or NF-κB activation is key proarrhythmic mediators of multiple pathophysiological signals in AF (5). We next determined whether the knockdown of Bhlhe40 could reduce the inflammation-related signaling pathways in Ang II-induced AF. Ang II-induced activation of p65 and NLRP3 signals in the atria of the rAAV9-shCON-injected mice was markedly reversed in the rAAV9-shBhlhe40-injected mice (Figure 6E). Neither group treated with saline showed significant differences in these parameters (Figures 6A–E).

## Discussion

**FIGURE 7:**
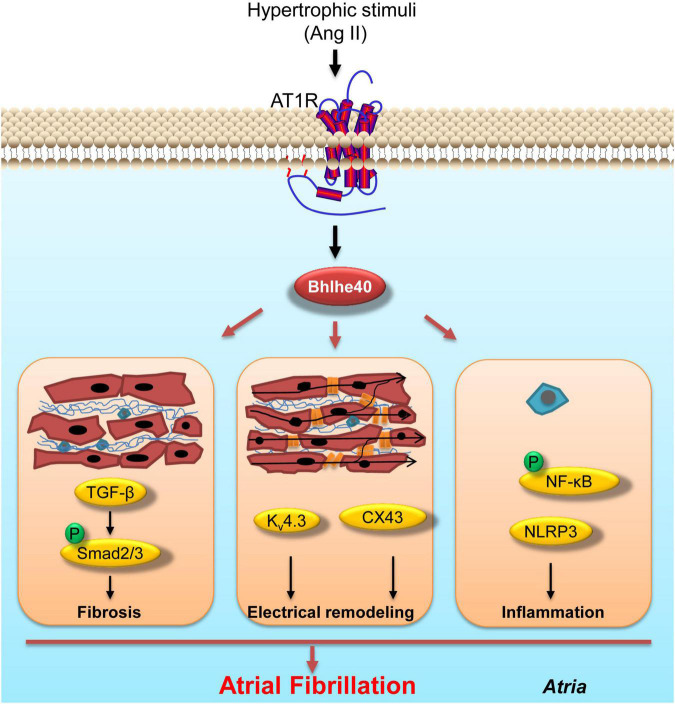
Working model of the effect of knockdown of Bhlhe40 on Ang II-induced AF. Cardiac-specific knockdown of Bhlhe40 suppresses Ang II-induced atrial inflammation, atrial fibrosis, electrical remodeling, and signaling pathways (NF-κB, NLRP3/IL-1β, TGF-β/Smad2, Kv4.3, and CX43) in mice.

In this study, for the first time, we demonstrated the role of Bhlhe40 in regulating Ang II-induced atrial fibrosis, atrial electrical remodeling, atrial inflammation, and the progression of AF. Bhlhe40 was significantly upregulated both in the serum of patients with AF and in atrial tissues from patients with AF. In a mice model of AF, the transcription and translation of Bhlhe40 were also upregulated by Ang II infusion. Functionally, atrial enlargement, susceptibility to atrial fibrillation, atrial fibrosis, atrial electrical remodeling, and atrial inflammation were found in the WT mice of Ang II infusion, whereas the loss of Bhlhe40 significantly attenuated Ang II-induced five-index changes. Mechanistically, we demonstrated that knockdown of Bhlhe40 blocked the activation of TGF-β/Smad2 and NF-κB/NLRP3/IL-1β signaling pathways, as well as the expression of CX43 and Kv4.3, in Ang II-infused mice. A working model is illustrated in Figure 7.

Growing evidence has revealed the role of the circadian rhythm genes in the development of cardiovascular diseases, such as hypertension, myocardial infarction, stroke, and AF (19, 20). Transcriptional factor Bhlhe40, also known as an important circadian rhythm factor, is a member of the Hairy/E(spl)/HES subgroup within the bHLH transcription factor family. Several studies reported that Bhlhe40 also plays a key role in cardiovascular diseases. For example, Bhlhe40 regulates the circadian rhythm of blood pressure through the transcriptional repression of ATP1B1 in the cardiovascular system (21). Systemic Bhlhe40 knockout protects the heart from pressure overload-induced fibrosis, inflammation, and myocardial cell apoptosis and improves the cardiac contractile function (22, 23). Interestingly, as we know, hypertension, LV hypertrophy, and congestive heart failure, induced by Ang II or pressure overload in mice models, predispose the mice to AF (24), suggesting that Bhlhe40 may be involved in the development of AF. Thus, we aim to investigate whether Bhlhe40 participated in hypertension/hypertrophic stimulus-induced atrial fibrosis, atrial inflammation, and the progression of AF and to confirm the underlying mechanism in Bhlhe40-knockdown mice.

Bhlhe40 is induced by various stress stimuli, such as hypoxia, tumor necrosis factor-α, irradiation, paclitaxel, and transforming growth factor-beta (TGF-β) (24), and participates in several pathogenic processes, including inflammation, apoptosis, tumor growth, and fibrosis (11, 25, 26). For example, Bhlhe40-deficient mice developed lymphoid organ hyperplasia and autoimmunity with age (8). Bhlhe40 deficiency inhibits the proliferation of CD4^+^ T cells and the production of interleukin (IL)-2, interferon-gamma (IFN-γ), and IL-4 (8). Bhlhe40 deficiency attenuates pulmonary fibrosis and repressed the PI3K/AKT/GSK-3β/β-catenin-integrated signaling pathway in mice and in A549 cells (27). These studies examined the canonical function of Bhlhe40 in immune cells or using systemic Bhlhe40-knockout mice, which could not distinguish the effect of Bhlhe40 in cardiomyocytes from that in other types of cells. Interestingly, our study demonstrated that Bhlhe40 is upregulated with Ang II infusion in atrial cardiomyocytes (Figures 2C,D). Similarly, previous studies reported that Bhlhe40 is upregulated not only in stromal cells but also in myocardial cells in TAC-induced hypertrophic heart tissues, suggesting that Bhlhe40 may play an important role in myocardial cells. Here, our study, using recombinant adeno-associated virus serotype 9 (rAAV9) to cardiac-specifically knock down Bhlhe40, is the first to demonstrate a direct relationship between the augmented expression of Bhlhe40 in atrial cardiomyocytes and patients with AF.

Inflammation, a common factor in a variety of cardiovascular diseases, increases the risk of AF and inflammatory cytokines, such as IL (interleukin)-1β, IL-6, GM-CSF (granulocyte–macrophage colony-stimulating factor), and TNF (tumor necrosis factor)-α, and is associated with atrial structural and electrical remodeling that triggers AF (28, 29). Inflammation is implicated in the pathophysiology of AF, and excessive inflammatory mediators diffuse into atrial tissue, altering its structural and electrical properties (30). Bhlhe40 also shows a potent pro-inflammatory effect. Bhlhe40 promotes inflammation and glycolysis in response to LPS *via* elevating the expression of HIF-1α in macrophages (31). Meanwhile, studies have reported that Bhlhe40 targets and inhibits the expression of IL-10, an anti-inflammatory cytokine, during *M. tuberculosis* infection (32). Our data demonstrated that the expression of two pro-inflammatory cytokines (IL-1β and IL-6 at the transcriptional level) and the extent of inflammatory cell infiltration (F4/80-positive cells), were decreased in cardiac-specific Bhlhe40-knockdown mice with Ang II infusion compare with the control mice with Ang II infusion, suggesting that Bhlhe40 may regulate Ang II-induced AF *via* regulating atrial inflammation. Recent studies demonstrate that cardiomyocyte (CM) NLRP3 (NACHT, LRR, and PYD domains-containing protein-3) inflammasome activation and NF-κB activation are key proarrhythmic mediators of multiple pathophysiological signals in AF and have direct effects on atrial fibrosis, ion channel, and connexin dysfunction in mouse and rabbit atria (4, 6, 7). For instance, it has been reported that mice with cardiomyocyte-restricted constitutive activation of the NLRP3 inflammasome have increased atrial ectopic activity and AF susceptibility, as well as enhanced mRNA expression of key ion channel subunits (RyR2, Kv1.5, GIRK1, and GIRK4) (6). In addition, the increased activation of NLRP3 inflammasomes can activate caspase-1 and promote the release of IL-1β and IL-18 (6). Moreover, NF-κB, an important transcriptional factor, has been implicated in the regulation of pro-inflammatory cytokines (IL-1β and IL-6) (33), ion channels (Kv4.3 and SCN5A), and gap junction proteins (CX43) (34). Our data showed that cardiac-specific knockdown of Bhlhe40 blocked the atrial inflammation and fibrosis caused by Ang II infusion and was accompanied by the inhibition of NF-κB activation, NLRP3/IL-1β, and TGF-β/Smad2 signaling. Meanwhile, the Ang II-induced increased expression of CX43 protein and decreased expression of Kv4.3 protein were reversed in the cardiac-specific knockdown of Bhlhe40 mice. Together, our data suggested that Bhlhe40 may involve in atrial remodeling and the progression of AF *via* regulating inflammatory signaling pathways, and inhibition of Bhlhe40 may be a potential novel anti-AF approach.

There are several limitations in our study. First, we confirmed an increased expression of Bhlhe40 in CMs of patients with AF and Ang II-infused mice, but we did not investigate the upstream factors that promote the expression of Bhlhe40 in the current study. Due to the limited access to atrial tissue samples we were unable to separate the nuclear fragments and cytoplasmic fragments from atrial tissues to assess the nuclear levels of Bhlhe40 in patients with AF, which the nuclear-translocation of Bhlhe40 has the activation of transcriptional suppressor promoted by SUMOylation (35). Thus, it is unclear whether post-translational modification, such as ubiquitination or SUMOylation, participated in regulating the expression or transcriptional activity of Bhlhe40 in patients with AF. In addition, because AAV9 is expressed in both atrial and ventricular CMs, the cardiac-specific knockdown of Bhlhe40 should affect both atrial and ventricular functions. Therefore, a loss of expression of Bhlhe40 in the atrium could be utilized to demonstrate that the increased atrial Bhlhe40 activity may be an underlying cause of AF development. In future, these important issues need to be well addressed.

In summary, our current research has revealed that the increased expression of Bhlhe40 in atrial tissues and cardiomyocytes both in patients with AF and in Ang II-induced mice plays an important role in the pathogenesis of AF. The cardiac-specific expression of Bhlhe40 is required for the progression of AF and the underlying atrial structure and electrical remodeling events. As far as we know, this study is the first to demonstrate a connection between cardiac Bhlhe40 and AF pathophysiology. Our results suggest that inhibition of the expression and/or transcriptional activity of Bhlhe40 prevents AF promotion and may be used as a novel approach to counteract AF by targeting both electrical and structural remodeling.

## Data availability statement

The original contributions presented in this study are included in the article/Supplementary material, further inquiries can be directed to the corresponding authors.

## Ethics statement

The studies involving human participants were reviewed and approved by the First Affiliated Hospital of Dalian Medical University (PJ-KS-KY-2021-229). The patients/participants provided their written informed consent to participate in this study. The animal study was reviewed and approved by Animal Care and Use Committee of Dalian Medical University.

## Author contributions

H-LB and H-HL conceived the project. K-WR, X-HY, Y-HG, XX, and YW performed the experiments and analyzed the data. K-WR, X-HY, and S-HW were responsible for human clinical studies and analyses. H-HL and H-LB wrote the article with input from all authors.

## Supplementary material

Supplementary Figure 1 The efficiency of knockdown of Bhlhe40 by rAAV9-shBhlhe40 injection in atria, lungs and skeletal muscles. The IHC staining of Bhlhe40 (left) and the quantification of the Bhlhe40-positive area (right) in **(A)** atria, **(B)** lungs, and **(C)** skeletal muscles from Ang II-infused or saline-infused mice (*n* = 6). Scale bar = 50 μm. **P* < 0.05 and ns = not significant.
