# Supplementary figures and images for: Cardiac-specific knockdown of Bhlhe40 attenuates angiotensin II (Ang II)-Induced atrial fibrillation in mice
Source: Front Cardiovasc Med. 2022 Oct 11;9:957903. doi: 10.3389/fcvm.2022.957903 (PMC9592817; doi:10.3389/fcvm.2022.957903)

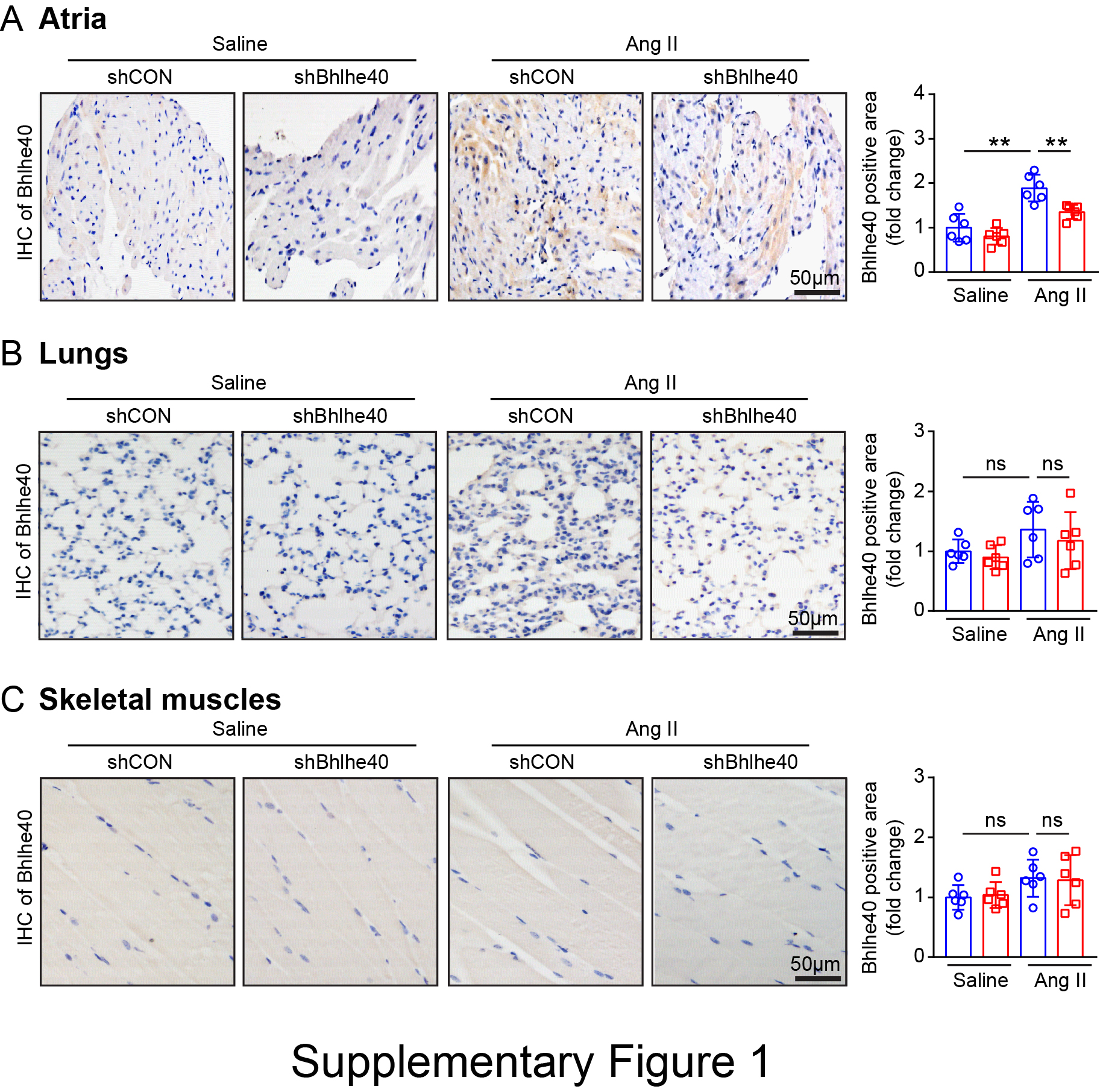

Supplement: Supplementary Figure 1 — The efficiency of knockdown of Bhlhe40 by rAAV9-shBhlhe40 injection in atria, lungs and skeletal muscles. The IHC staining of Bhlhe40 (left) and the quantification of the Bhlhe40-positive area (right) in (A) atria, (B) lungs, and (C) skeletal muscles from Ang II-infused or saline-infused mice (n = 6). Scale bar = 50 μm. *P < 0.05 and ns = not significant. [file Image_1.JPEG]
